# Antitrypanosomal, Antitopoisomerase-I, and Cytotoxic Biological Evaluation of Some African Plants Belonging to Crassulaceae; Chemical Profiling of Extract Using UHPLC/QTOF-MS/MS

**DOI:** 10.3390/molecules27248809

**Published:** 2022-12-12

**Authors:** Mostafa M. Hegazy, Wael M. Afifi, Ahmed M. Metwaly, Mohamed M. Radwan, Muhamad Abd-Elraouf, Ahmed B. M. Mehany, Eman Ahmed, Shymaa Enany, Shahd Ezzeldin, Adel E. Ibrahim, Sami El Deeb, Ahmad E. Mostafa

**Affiliations:** 1 Department of Pharmacognosy and Medicinal Plants, Faculty of Pharmacy, Al-Azhar University, Cairo 11884, Egypt; 2 Department of Pharmacognosy, Faculty of Pharmacy, Sinai University—Kantara Branch, Ismailia 41636, Egypt; 3 National Center for Natural Products Research, University of Mississippi, University, MS 38677, USA; 4 Department of Pharmacognosy, Faculty of Pharmacy, University of Alexandria, Alexandria 21521, Egypt; 5 Zoology Department, Faculty of Science, Al-Azhar University, Cairo 11884, Egypt; 6 Department of Pharmacology, Faculty of Veterinary Medicine, Suez Canal University, Ismailia 41522, Egypt; 7 Proteomics and Metabolomics Research Program, Department of Basic Research, Children’s Cancer Hospital 57357, Cairo 11441, Egypt; 8 Department of Microbiology and Immunology, Faculty of Pharmacy, Suez Canal University, Ismailia 41522, Egypt; 9 Natural and Medical Sciences Research Center, University of Nizwa, Birkat Al Mauz, P.O. Box 33, Nizwa 616, Oman; 10 Pharmaceutical Analytical Chemistry Department, Faculty of Pharmacy, Port-Said University, Port-Said 42511, Egypt; 11 Institute of Medicinal and Pharmaceutical Chemistry, Technische Universitaet Braunschweig, 38106 Braunschweig, Germany

**Keywords:** Crassulaceae, antitrypanosomal, cytotoxic, UHPLC/QTOF-MS, phenolics, flavonoids

## Abstract

In our continuous study for some African plants as a source for antitrypanosomally and cytotoxic active drugs, nine different plants belonging to the Crassulaceae family have been selected for the present study. *Sedum sieboldii* leaves extract showed an antitrypanosomal activity against *Trypanosoma brucei* with an IC_50_ value of 8.5 µg/mL. In addition, they have cytotoxic activities against (HCT-116), (HEPG-2) and (MCF-7), with IC_50_ values of 28.18 ± 0.24, 22.05 ± 0.66, and 26.47 ± 0.85 µg/mL, respectively. Furthermore, the extract displayed inhibition against Topoisomerase-1 with an IC_50_ value of 1.31 µg/mL. It showed the highest phenolics and flavonoids content among the other plants’ extracts. In order to identify the secondary metabolites which may be responsible for such activities, profiling of the polar secondary metabolites of *S. sieboldii* extract via Ultra-Performance Liquid Chromatography coupled to High-Resolution QTOF-MS operated in negative and positive ionization modes, which revealed the presence of 46 metabolites, including flavonoids, phenolic acids, anthocyanidins, coumarin, and other metabolites.

## 1. Introduction

Trypanosomiasis is a devastating African disease called sleeping sickness that is caused by the *Trypanosome brucei* parasite. Tsetse flies, which are prevalent along the geographical sub-Saharan Africa [1], are the main transmitters of this disease. African plants reported as rich sources for antitrypanosomal drugs [2,3]. Meanwhile, cancer can be considered the second global cause of death, with an average rate of 1 per 6 deaths and a total of 9.6 million deaths, as estimated in 2018 [4]. Several plant species have been reported to prevent the development of cancer or even used as a cancer treatment, with plant derived compounds demonstrating inhibitory effects on cancer cell activity through the inhibition of proliferation of cancer cells and inducing apoptotic cell death [5]. In Folk Medicine, herbalists and botanists in North African countries prescribe several plants for cancer treatment [6]. Many plants belonging to the Crassulaceae family were reported to have antileishmanial activity [7,8], antiprotozoal activities [9,10], as well as anticancer activities [11]. The dual activities of natural sources and/or synthetic compounds as antiprotozoal and cytotoxic agents had been also reported [12]. Furthermore, the inhibition of the histone deacetylase (HDAC) enzyme had been reported as a target for this dual effect [12]. Topoisomerases are important nuclear enzymes that play a vital role in DNA replication, transcription, chromosome segregation, and recombination [13]. Topoisomerase I (Topo I) and topoisomerase II (Topo II) are the main two types of topoisomerases. The responsibility of cleavage of DNA duplex, relaxing, and release is due to Topo-I, while Topo-II is responsible for cleaving the DNA helix simultaneously to remove DNA supercoiling [14]. Therefore, topoisomerases are recently targeted by newly developed cancer chemotherapeutics [15]. Topoisomerase inhibitors generate single and double-stranded breaks by blocking the cell cycle’s ligation step that, in turn, harms the integrity of the genome [16]. A naturally occurring camptothecin was reported to have a topoisomerase I inhibition activity against trypanosomes and leishmania [17]. In addition, the naturally occurring camptothecin and rebeccamycin were found to inhibit the activity of topoisomerase I, causing an arrest of the proliferation of cancer cells and *Trypanosoma cruzi* [18]. Furthermore, several synthetic compounds showed both antiprotozoal and cytotoxic activities [19]. Crassulaceae is a family of 34 genera and 1410 species distributed worldwide [20]. The crassulaceae family contains different types of secondary metabolites, such as flavonoids [21], tannins [22], bufadienolides [23], alkaloids [22], triterpenes [24], and sterols [25]. Phenolics are widely distributed secondary metabolites in the plant kingdom and take great attention due to their promising biological activities [26,27]. Gallic acid (3,4,5-trihydroxybenzoic acid), a naturally abundant plant phenolic compound, showed promising activity against *Trypanosoma brucei* with LD_50_ value of 46.96 ± 1.28 µM [28]. A research study suggested that the pyrogallol moiety could be responsible for the antitrypanosomal activity [28]. Some hydroxyflavones showed promising antitrypanosomal activities with IC_50_ values less than 0.5 μg/mL [29]. Luteolin and quercetin were reported as active against *T. cruzi* with IC_50_ values of 0.8 and 1.0 μg/mL, respectively [29]. 

Being very rich in poly phenolics [21,22], the Crassulaceae family inspired the authors to select several plants belonging to this family, calculate their phenolics and flavonoids contents, examine their antitrypanosomal, antiprotozoal, and cytotoxic activities, and then to find out the correlation between their chemical composition and the resulted biological activities. 

In the proposed study, nine plants belonging to family Crassulaceae have been extracted with 70% ethanol, and these extracts were examined for their antitrypanosomal, antilishmanial, antimalarial, and cytotoxic activities. Moreover, the total phenolic content (TPC) and total flavonoids contents (TFC) were assessed for all extracts. Then, the most active antitrypanosomal extract and the one with the highest phenolic and flavonoidal content was selected to evaluate its Topoisomerase I inhibition activity. This extract was also identified for the nature of its major secondary metabolites and was identified using ultra-high performance liquid chromatography coupled with quadrupole time of flight mass spectrometry (UHPLC/QTOF-MS), which may be responsible for these promising activities.

Another aim for the proposed research was trying to figure out the biological importance of different African plants belong to the Crassulaceae family. The obtained findings urged the authors to examine both the cytotoxic activities of plant extracts against Human Colon Carcinoma (HCT-116), Human Hepatocyte carcinoma (HEPG-2), and Human Breast adenocarcinoma (MCF-7) cell lines, besides the topoisomerase I inhibition activity for the most active extract among the species of this study.

## 2. Results and Discussions

### 2.1. Total Phenolic Content (TPC) and Total Flavonoids Contents (TFC) Assay

The total phenolic content (TPC) and total flavonoids contents (TFC) for all plants were processed to find out if there is a relation between the phenolic content and activity. Interestingly, *S. sieboldii* exhibited the highest amounts of total phenolic and total flavonoid contents with concentrations of 170.1 mg gallic acid equivalents (GAE)/100 gm and 40.2 mg quercetin equivalents (QE)/100 gm, respectively. Table 1 shows the total flavonoidal and phenolic contents of the extracts of the plants under study. 

### 2.2. Antitrypanosomal Examination

Nine plants belonging to Crassulaceae family have been extracted. The extracts were examined for their antitrypanosomal. The *Sedum sieboldii* leaves ethanolic extract is the only one that exhibited a promising activity against *Trypanosoma brucei* with an IC_50_ value of 8.55 µg/mL. This result could emphasize the relationship between the phenolic and flavonoidal content and the antitrypanosomal activity (Table 1).

### 2.3. Cytotoxic Examination

The examination of the cytotoxic activities of plant extracts was driven by their well proven relation between antiprotozoal and cytotoxic activities. The cytotoxic activities of plant extracts were examined against human hepatocyte carcinoma (HEPG-2), human breast adenocarcinoma (MCF-7), and human colon carcinoma (HCT-116) cell lines. Several plant extracts (including *S. sieboldii*) exhibited promising cytotoxic activities (Table 2).

### 2.4. Topo I Inhibitory Activity

The most active antitrypanosomal extract (*Sedum sieboldii*) was evaluated for its inhibitory activity against the Topo I enzyme using staurosporine as a positive control in this procedure. *Sedum sieboldii* ethanolic extract displayed inhibitory activity for topoisomerase I (Topo I) with an IC50 value of 1.31 µg/mL.

### 2.5. LC–MS/MS Assay

The use of chromatography in identification and separation of analytes has played an important role within the past decades, where several sensitive detectors and separation modes were being developed [30]. UHPLC/QTOF-MS is a useful technique that, when operated in the negative and positive ionization modes as non-targeted profiling method [31], can identify the analytes through precisely measuring their ionic mass and its fragmentation patterns. The most active antitrypanosomal extract (*Sedum sieboldii*) was analyzed using electrospray ionization (ESI-MS) in positive and negative-ion modes to avoid any change in competitive ionization and suppression effects due to the changes in ESI polarity which can often circumvent or significantly alter, revealing otherwise suppressed metabolite signals.

**Figure 1:**
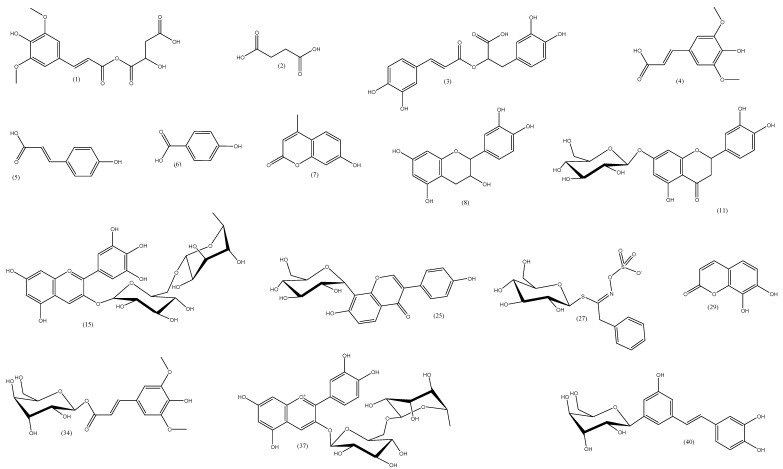
Compounds tentatively identified by UHPLC/QTOF–MS of *S. sieboldii* extract.

In total, 30 peaks from *S. sieboldii* ethanolic extract were identified based on their negative-ionization mass spectral data versus 10 in the positive-ion mode (see Figure 1 and Table 3 and Table 4). A total of 40 secondary metabolites were detected and identified. Metabolites belonged to several natural product classes including 27 flavonoids, 7 phenolic acids, 2 coumarins, 2 anthocyanidins, 1 stilbene, 1 dicarboxylic acid, and 1 glucosinolate.

#### 2.5.1. Identification of Flavonoids

The fragmentation behaviors of most flavonoids yielded prominent [M-H]^−^ ions [32]. They tended to lose CO, H_2_O, and CH_3_ due to the existence of phenolic, hydroxyl, and methyl as subgroups attached to the flavonoid aglycon [32]. The sugars moieties attached to the flavonoid aglycon could be detected due to their losses in the MS analysis, and could be represented as [M-H-146]^−^, [M-H-132]^−^, [M-H-162]^−^, and [M-H-176]^−^, for the loss of rhamnose, pentose, hexose, and hexuronic acid moieties, respectively [33].

**Table 4:** Compounds tentatively identified by mass spectra of *S. sieboldii* extract, continued.

P	Tentative Assignment	RT (min)	Chemical Formula	Precursor m/z	MS [−] MS/MS m/z	MS [+] MS/MS *m/z*	Error (ppm)	Ref.
1	Sinapoyl malate	0.446	C_15_H_16_O_9_	339.0567	339.0567 [M-H]^−^, 223.04 [M-H-malate]^−^		0.3	[34]
2	Succinic acid	0.533	C_4_H_6_O_4_	116.9861	116.9861 [M-H]^−^, 73.03 [M-H-CO_2_]^−^		0	[35]
3	Rosmarinic acid	0.751	C_18_H_16_O_8_	359.0998	359.0998		1.6	[36]
4	Sinapic acid	0.791	C_11_H_12_O_5_	223.046	223.046 [M-H]^−^		1	[37]
5	P-coumaric acid	0.967	C_9_H_8_O_3_	163.0402	163.0402 [M-H]^−^, 119.05 [M-H-COOH]^−^		1	[37]
6	P-hydroxybenzoic acid	1.032	C_7_H_6_O_3_	137.0245	137.0245 [M-H]^−^, 93.03 [M-H-CO_2_]^−^		0.8	[37]
7	7-hydroxy-4-methylcoumarin (Hymecromon)	1.237	C_10_H_8_O_3_	175.0974	175.0974 [M-H]^−^, 130.97 [M-H-CO_2_]^−^		2.3	[38]
8	(+) Epicatechin	1.715	C_15_H_14_O_6_	289.1311	289.1311 [M-H]^−^		−5.7	[37,39]
9	Kaempferol-7-neohesperidoside	1.731	C_27_H_30_O_15_	593.1486	593.1486 [M-H]^−^, 447.68 [M-H-Rhamnosyl]^−^		2.3	[40]
10	Luteolin-6-C-glucoside (Isoorientin)	2.841	C_21_H_20_O_11_	449.1082		449.1082 [M+H]^+^	−0.2	[41]
11	Eriodictyol-7-o-glucoside	3.071	C_21_H_22_O_11_	449.0722	449.0722 [M-H]^−^, 287.02 [M-glucose-H]^−^		0.7	[42]
12	Apigenin-6-C-glucoside -7-O-glucoside (Saponarin)	3.079	C_27_H_30_O_15_	595.1672		595.1672 [M+H]^+^	−0.7	[43]
13	Myricitrin	3.189	C_21_H_20_O_12_	463.0877	463.0877 [M-H]^−^		0.9	[44]
14	Vitexin-2″-o-rhamnoside	3.213	C_27_H_30_O_14_	577.1559	577.1559 [M-H]^−^, 457.11, 413.07, 293.05		0.5	[45]
15	Delphinidin-3-o-(6″-o-alpha-rhamnopyranosyl-β-glucopyranoside)	3.333	C_27_H_31_O_16_	609.1464	609.1464 [M-2H]^−^		−0.3	[46]
16	Apigenin 8-c-glucoside (vitexin)	3.386	C_21_H_20_O_10_	431.0981	431.0981 [M-H]^−^		0	[41]
17	Syringetin-3-o-galactoside	3.614	C_23_H_24_O_13_	507.1167	507.1167 [M-H]^−^		−3.2	[47]
18	Kaempferol-3-glucuronide	3.688	C_21_H_18_O_12_	461.1086	461.1086 [M-H]^−^		0.3	[48]
19	Quercetin-3-d-xyloside (Reynoutrin)	3.745	C_20_H_18_O_11_	433.0757	433.0757 [M-H]^−^		4.3	[49]
20	Baicalein-7-o-glucuronide (Baicalin)	3.862	C_21_H_18_O_11_	445.1104	445.1104 [M-H]^−^		0.8	[50]
21	Kaempferol-3,7-O-bis-α-L-rhamnoside (Kaempferitrin)	4.043	C_27_H_30_O_14_	579.1714		579.1714 [M+H]^+^	0.3	[51,52]
22	Quercetin-7-o-rhamnoside	4.105	C_21_H_20_O_11_	447.0931	447.0931 [M-H]^−^		0	[39]
23	Rhoifolin	4.389	C_27_H_30_O_14_	577.1563	577.1563 [M-H]^−^		−0.2	[53]
24	Myricetin	4.463	C_15_H_10_O_8_	317.0298	317.0298 [M-H]^−^		1.6	[52,54]
35	Daidzein-8-c-glucoside (Puerarin)	4.774	C_24_H_40_O_5_	415.196	415.196 [M-H]^−^		0.9	[55]
26	Luteolin	5.592	C_15_H_10_O_6_	285.0396	285.0396 [M-H]^−^		2.8	[45,52]
27	Benzyl glucosinolate	5.793	C_14_H_19_NO_9_S_2_	408.1458	408.1458 [M-H]^−^		−5.1	[56]
28	Quercetin	5.835	C_15_H_10_O_7_	303.0484		303.0484 [M+H]^+^	1.2	[45]
29	7, 8-Dihydroxycoumarin (Daphnetin)	6.006	C_9_H_6_O_4_	177.0553	177.0553 [M-H]^−^		1.4	[57]
30	Apigenin	6.519	C_15_H_10_O_5_	269.0456	269.0456 [M-H]^−^		0.4	[45,52]
31	3 5 7-trihydroxy-4′-methoxyflavone (Kaempferide)	6.786	C_16_H_12_O_6_	299.0561	299.0561 [M-H]^−^		−0.3	[58]
32	Kaempferol-3-o-α-l-arabinoside	7.333	C_20_H_18_O_10_	417.1781	417.1781 [M-H]^−^		−3.3	[59]
33	Kaempferol-3-o-α-l-rhamnoside (kaempferin)	9.919	C_21_H_20_O_10_	431.1713	431.1713 [M-H]^−^		−0.5	[52,60]
34	1-O-β-D-glucopyranosyl sinapate	9.921	C_17_H_22_O_10_	387.1808		387.1808 [M+H]^+^	−0.9	[61]
35	Gossypin	11.194	C_21_H_20_O_13_	481.1479		481.1479 [M+H]^+^	0.6	[62]
36	Quercetin-4′-glucoside	11.271	C_21_H_20_O_12_	465.1524		465.1524 [M+H]^+^	0.2	[63]
37	cyanidin-3-O-rutinoside	11.872	C_27_H_31_O_15_	595.2518		595.2518 [M]^+^	0.7	[64]
38	Luteolin-8-C-glucoside	12.651	C_21_H_20_O_11_	449.1575		449.1575 [M+H]^+^	0.4	[41]
39	Acacetin-7-O-neohesperidoside (Fortunellin)	12.885	C_28_H_32_O_14_	593.2344		593.2344 [M+H]^+^	1.7	[65]
40	E-3,4,5′-trihydroxy-3′-glucopyranosylstilbene (Astringin)	13.795	C_20_H_22_O_9_	405.2042	405.2042 [M-H]^−^		2.2	[66]

Flavonoids in *S. sieboldii* extract represented the most abundant metabolites and showed twenty-nine peaks, represented by different flavonoid classes (Table 4). Flavone subclasses were tentatively identified due to the presence of fifteen peaks, with their characteristic parent ion peaks in positive and negative ion modes at 609.1502 [M-H]^−^, 449.1082 [M+H]^+^, 285.0396 [M-H]^−^, 449.1575 [M+H]^+^, corresponding to luteolin and its glycosides (Table 4); while the characteristic parent ion peaks in positive and negative ion modes at 595.1672 [M+H]^+^, 577.1559 [M-H]^−^, 431.0981 [M-H]^−^, 577.1563 [M-H]^−^, 269.0456 [M-H]^−^, 593.2344 [M+H]^+^, corresponding to apigenin and its glycosides (Table 4); and the characteristic parent ion peaks in negative ion mode at 463.0877 [M-H]^−^, 507.1167 [M-H]^−^, 317.0298 [M-H]^−^, corresponding to myricetin and its glycosides (Table 4); and the parent ion peak at 445.1104 [M-H]^−^, corresponding to Baicalein-7-o-glucuronide; and the parent ion peak at 481.1479 [M+H]^+^, corresponding to gossypin. Flavonol subclasses were tentatively identified due to the presence of eleven peaks with their characteristic parent ion peaks in positive and negative ion modes at 593.1486 [M-H]^−^, 461.1086 [M-H]^−^, 579.1714 [M+H]^+^, 299.0561 [M-H]^−^, 417.1781 [M-H]^−^, 431.1713 [M-H]^−^, corresponding to kaempferol and its glycosides (Table 4); while the characteristic parent ion peaks in positive and negative ion modes at 433.0757 [M-H]^−^, 447.0931 [M-H]^−^, 447.0998 [M-H]^−^, 303.0484 [M+H]^+^, corresponding to quercetin and its glycosides (Table 4). Flavanol, flavanone, and isoflavonoids’ subclasses were tentatively identified due to the presence of their characteristic parent ion peaks in negative ion modes at 289.1311 [M-H]^−^, 449.0722 [M-H]^−^, 415.196 [M-H]^−^, corresponding to epicatechin, eriodictyol-7-*o*-glucoside, and daidzein-8-*c*-glucoside, respectively (Table 4).

Kaempferol previously reported as antiprotozoal [67], as well as the antileishmanial activity that had been reported for quercetin, kaempferol, and kaempferol glycoside [68,69]. Furthermore, quercetin proved to induce apoptosis of *T. brucei* [70]. In fact, different flavonoids exhibited antiprotozoal activities [29]. Additionally, different flavonoids exhibited good activities against different species of *Leishmania* with IC_50_ values in the range of 16.6 to 52 μg/mL [71,72]. 

The well documented antiprotozoal activities of flavonoids aside, with the high flavonoid contents of *S. sieboldii* ethaolic extract, suggested that its antitrypanosomal activity may be linked to its flavonoidal content.

#### 2.5.2. Phenolic Acids Identification

Phenolic acids generally showed its parent ion [M-H]^−^ in the negative mode corresponding to the deprotonated molecule, as well as the characteristic fragment ion [M-H-44]^−^ corresponding to the loss of CO_2_ from the carboxylic acid group. Phenolic acids tentatively identified in *S. sieboldii* as sinapoyl malate with its characteristic peaks in negative ion mode at 339.0567 [M-H]^−^ and at 223.04 [M-H-malate]^−^, rosmarinic acid with its characteristic peak at 359.0998 [M-H]^−^ in negative ion mode, Sinapic acid with its characteristic peak in negative ion mode at 223.046 [M-H]^−^, Caffeic acid with its characteristic peaks in negative ion mode at 179.0376 [M-H]^−^ and 135.04 [M-H-COOH]^−^, *p*-coumaric acid with peaks in negative ion mode at 163.0402 [M-H]^−^ and 119.05 [M-H-COOH]^−^, P-hydroxybenzoic acid with peaks in negative ion mode at 137.0245 [M-H]^−^ and 93.03 [M-H-CO_2_]^−^, 1-O-b-D-glucopyranosyl sinapate with a peak in positive ion mode at 387.1808 [M+H]^+^.

#### 2.5.3. Other Metabolites

The other secondary metabolites tentatively identified in *S. sieboldii* represented different classes as coumarins, such as 7-hydroxy-4-methylcoumarin, 6,7-dihydroxycoumarin, and 7,8-dihydroxycoumarin, and their parent ion peaks in negative ion mode due to loss of hydrogen were 175.0974, 177.0179, and 177.0553, respectively. Also, alkaloids were represented and tentatively identified as trigonelline and nicotine, and their parent ion peaks in positive ion mode were 138.0527 [M+H]^+^ and 163.0582 [M+H]^+^. Other metabolites tentatively identified were two anthocyanidins: Delphinidin-3-o-(6′′-o-alpha-rhamnopyranosyl-beta-glucopyranoside) with ion peak at 609.1464 [M-2H]^−^, cyanidin-3-O-rutinoside with ion peak at 595.2518 [M]^+^ and a stilbene; astringin with an ion peak at 405.2042 [M-H]^−^ one dicarboxylic acid; Succinic acid with ion peaks at 116.9861 [M-H]^−^, 73.03 [M-H-CO_2_]^−^ and a glucosinolate; Benzyl glucosinolate with an ion peak at 408.1458 [M-H]^−^.

## 3. Materials and Methods

### 3.1. Plant Materials and Extraction

*Crassula erosula*, *Crassula ovata*, *Crassula convolute*, *Crassula obliqa*, *Crassula mesembryanthemoides*, *Crassula portulacaria*, *Sedum anacampseros*, *Sedum sieboldii*, and *Sedum nussbaumerianum* were collected and identified by the Botanical team of Al-Orman Botanical Garden, Giza, Egypt in January 2017. Voucher specimens (C-171 to C-176, and S-171 to S-173) have been deposited in Al-Azhar University, Faculty of Pharmacy (Pharmacognosy Department) in Cairo, Egypt. Samples of 10 g fresh leaves were prepared for extraction through cutting by mixer. Then, the samples were exhaustively extracted with 70% ethanol sonicated at 30 kHz for 60 min. The samples were then filtered. The marc was re-extracted 3 times as described above. The collected extracts were combined, filtrated, and dried under reduced pressure at 40 °C.

### 3.2. Total Phenolic Contents

The total phenolic content (TPC) of the plant extract was assessed by UV-Visible spectrophotometer (UV Analyst-CT 8200) using gallic acid as standard, according to the Folin–Ciocalteau method [73]. A standard calibration curve for gallic acid was constructed within the range of 10–50 against absorbance, at a wavelength of 765 nm. Each 1 mL plant extract sample was diluted with 5 mL Folin-Ciocalteu’s reagent (1:10 diluted in water) and 4 mL sodium carbonate solution (7.5%, *w/v* in water). Each sample was repeated twice, and diluted samples were left at 25 °C for 60 min. The samples’ absorbances were then measured at 765 nm and the concentration of TPC was calculated from gallic acid calibration curve as gallic acid equivalents (GAE) in mg/100 fresh weight (f.w.).

### 3.3. Total Flavonoid Contents

UV-VIS spectrophotometry was used for determination of the total flavonoidal content using a UV-Analyst double beam spectrophotometer (model-CT 8200) and measured according to aluminum chloride colorimetric methods [74]. The total flavonoid content was in terms of quercetin equivalents (QE) mg/100 g fresh weight (f.w.). Serial concentrations of quercetin standard solutions were prepared and 1 mL was added to each 5.0 mL distilled water and 0.3 mL of sodium nitrite (5%, *w/v* in water). Then, after exactly 5 min, 0.3 mL of aluminium chloride solution (10%, *w/v* in water) was added. After another 2 min, 2 mL of sodium hydroxide solution (1 M) were added to the mixture, mixed well, and then immediately brought to 10 mL standard volume with water. The absorbance was measured at 510 nm. The blank experiment was performed and each sample was analyzed in the same way in duplicate.

### 3.4. Assay of Antitrypanosomal Activity

A culture of *Trypanosoma brucei* cultured for 2 days (exponential phase) was used. It was first diluted with IMDM medium (5000 parasites per mL). DMSO had a maximum permissible limit of 0.5% and the assay was performed using clear 96-wells microplates. Stock extracts were diluted from 20 mg/mL for primary screening to dilutions of 1 mg/mL in IMDM medium. A single concentration of 20 μg/mL in duplicate. A sample of 4 μL of each extract dilution was placed in each well together with the culture (196 μL). Microplates were incubated for 48 h in 5% carbon dioxide at temperature of 37 °C. Another 10 μL of Amar blue was added (AbD Serotec, Oxford, UK; cat. no.; BUF012B) to each microplate well, then they were incubated further overnight. BMG FLUOstar Galaxy microplate reader from BMG LabTechn (Ortenberg, Germany) was used to measure the standard fluorescence at an excitation wavelength of 544 nm and emission of 590 nm. α-difluoromethylornithine and pentamidine were used as testing standards. Primary screening results indicated that the extracts showed +90% inhibition for the growth of *T. brucei*. Therefore, a secondary dose/ growth inhibition response analysis screening was performed at a range of 0.4–10.0 μg/mL concentrations of plant extracts. The values of IC_90_ and IC_50_ were computed using XLfit version 5.2.2 from the dose/growth inhibition response curve [75].

### 3.5. Cytotoxic Assay

Three cancer cell lines were screened for the anti-proliferative activity of the plant extract, namely human hepatocyte carcinoma (HEPG-2), human breast adenocarcinoma (MCF-7), and human colon carcinoma (HCT-116) cell lines. Quantitative measurement of the anti-cancer activity was performed using the protocol reported by Borenfreund and Puerner [76]. In this neutral red assay protocol, DMEM media (Lonza, Basel, Switzerland) was used to culture the cell lines which were supplemented with L-glutamine (0.2 M) and fetal bovine serum (10%), Gibco-BRL (Waltham, MA, USA). Dimethyl sulfoxide and DMEM mixture (at ratio 4/100, *v*/*v*) were used to dissolve the test compounds. The cell lines were tested using an initial dose of 1 mg/mL, which was followed by 7 serial dilutions for the dose (at 50% diluting factor) from the initial start dose. A concentration of 60,000 cells/mL of cells was seeded for 24 h in a 96-wells plate that was flat bottomed in conditions of 37 °C and carbon dioxide (5%) until obtaining a semi confluent cell layer. Then, the cell lines were treated with 100 µL of each dilution prepared serially of the test compounds. The anticancer activities were quantitatively assessed after 48 h using ELISA microplate readerset at 540 nm under the mentioned protocol [76]. 

### 3.6. Topo I Assay

Topo I assay was performed using the sandwich-based enzyme linked immune sorbent technique (item Specification; 48T/96T). Anti-TOP-I were first costed onto the 96-wells plate. The detection was done by biotin conjugated anti-TOP-I. In a sequence, standards were added, the test followed by detection anti-TOP-I, and finally wells were washed using the washing buffer. HRP Streptavidin was then placed and the washing buffer was used to remove unbound conjugates. Visualization of the HRP enzyme reaction was done using the TMB substrates, which were catalyzed by HRP. This led to the formation of blue colored product turning yellow following the addition of the acidic stop solution. The amount of TOP-I captured in the sample was directly proportional to the intensity of the yellow color, where the absorbance of the O.D. was measured in microplate reader at 450 nm. Finally, the TOP-I concentration was calculated [77].

### 3.7. LC–MS/MS

#### 3.7.1. Chemicals

Acetonitrile, ethyl acetate, and methanol were all HPLC grades and were purchased from Thermo-Fisher (Waltham, MA, USA). Isopropanol and dichloromethane were of analytical grades and were also purchased from Thermo-Fisher (Waltham, MA, USA). Ammonium formate, formic acid, ammonium acetate, and ammonium hydroxide were all of analytical grades which were purchased from Sigma-Aldrich (St. Louis, MO, USA).

#### 3.7.2. Sample Preparation

50 mg of the lyophilized plant extract were reconstituted in 1 mL of the reconstitution solvent. The reconstitution solvent was a mixture of water: methanol: acetonitrile (2:1:1, *v*/*v*/*v*). The reconstituted sample was vortex mixed for 2 min and then put in ultra-sonic for 10 min (20–30 kHz). Another 1 mL from the reconstitution solvent was added to the sample before centrifugation at 10,000 rpm for 5 min. The clear supernatant solution (concentration 1.0 µg/mL) was then used for chromatographic analysis where 10 µL were injected for a chromatographic HPLC/QTOF run in positive and negative modes. Blank and quality control samples were carried out in the same way. 

#### 3.7.3. Instruments and Acquisition Method

The ExionLC AC system was used for chromatographic separation from AB SCIEX (Vaughan, ON, Canada). The system consisted of a HPLC pump, auto-sampling part, column compartment, and was connected to QTOF-MS/MS detector Mass, model Triple TOF 5600+^®^ using Duo-Spray^®^ electrospray ionization (ESI) mode. Chromatographic separations were done using C18-RP column XSelect HSS T3 (2.5 µm, 2.1 × 150 mm) from Water (Milford, MA, USA). Mobile phase was eluted in the gradient technique at flow rate 0.3 mL/min and composed of mobile phase part-A and part-B. Part-A was 5 mM ammonium formate solution in water containing 1%, *v*/*v*, methanol and pH was adjusted to 3.0 using formic acid. Part-B was HPLC grade acetonitrile. The gradient program was set at start at 10% mobile phase part-B from 0–20 min. Then, part-B was increased to 90% from 21–25 min and finally to 10% again from 25–28 min. Colum temperature was set at 40 °C. The sprayer capillary and declustering potential ion-spray voltage floating were operated in positive and negative modes at voltages +4500/+80 and − 4500/− 80 V, respectively. The source temperature was set at 600 °C, the curtain gas was 25 psi, and gas 1 and gas 2 were 40 psi. The collision energies were +35 and −35 eV for positive and negative modes, respectively, and ion tolerance was 10 ppm. MS and MS/MS data were collected and analyzed using information-dependent acquisition (IDA) using Analyst TF 1.7.1. from AB SCIEX (Vaughan, ON, Canada). MS was operated in 50-ms pattern scans from 50–1100 *m/z* and the most intense ions were selected for acquiring MS/MS fragmentation spectra after each scan [78].

#### 3.7.4. Data Processing

The non-targeted analysis of small molecules was comprehensively carried out using MS-DIAL 3.70 Yokohama software from RIKEN Center for Sustainable Resource Science (Tsurumi-ku, Kanagawa, Japan), ReSpect-positive and ReSpect-negative databases (comprising 2737 and 1573 records, respectively). UHPLC/QTOF-MS is a very powerful technique that enables the tentative identification of unknown compounds by predicting the chemical formula using the accurately measured ion mass and its characteristic isotopic patterns [79]. Therefore, Koyoto Encyclopedia of Genes and Genomes (KEGG) [80] was used to retrieve and analyze the identified compounds in order to investigate the different molecules in the plant metabolic pathways.

## 4. Conclusions

Nine different plants of Crassula and Sedum species have been selected for screening of their anti-trypanosomal and anticancer activity, as well as determination of their phenolics and flavonoids contents. Among the nine plants of the proposed study, *Sedum sieboldii* total extract exhibited the highest contents of total phenolics and flavonoids. The extract showed an anticancer activity and a promising antitrypanosomal activity against *Trypanosoma brucei* with an IC_50_ value of 8.55 µg/mL. Furthermore, UHPLC/QTOF-MS was applied for such extract to tentatively identify its chemical constituents that could be a lead to such activities. These interesting results open the door for further research aiming at the development of a successful treatment for Trypanosoma from *Sedum sieboldii.*

## Figures and Tables

**Table 1:** Total phenolic and total flavonoid contents of the examined plants.

Plant Name	Total Phenolics (mg GAE/100 gm Fresh Weight)	Total Flavonoids (mg QE/100 gm Fresh Weight)
*C. convolute*	113.1 ± 4.8	23.6 ± 1.7
*C. erosula*	117.6 ± 5.8	25.9 ± 1.4
*C. mesembryanthemoides*	130.2 ± 6.2	28.2 ± 1.8
*C. obliqa*	89.5 ± 3.9	25.2 ± 2
*C. ovata*	110.3 ± 6.1	30.4 ± 1.8
*C. portulacaria*	114.2 ± 4.1	29.3 ± 1.9
*S. anacampseros*	139.9 ± 7.6	33.5 ± 2.1
*S. nussbaumerianum*	150.2 ± 5.9	35.4 ± 2.3
*S. sieboldii*	170.1 ± 9.1	40.2 ± 2.7

**Table 2:** IC_50_ (µg/mL) cytotoxic activities of the examined plants’ extracts.

Plant Name	HCT-116 Human Colon Carcinoma	HEPG-2 Human Hepatocyte Carcinoma	MCF-7 Human Breast Adenocarcinoma
*C. convolute*	12.05 ± 1.82	13.25 ± 1.14	11.15 ± 0.87
*C. erosula*	16.51 ± 0.68	15.96 ± 1.08	17.24 ± 0.36
*C. mesembryanthemoides*	15.16 ± 0.18	10.33 ± 0.78	13.45 ± 0.76
*C. obliqa*	37.44 ± 0.38	38.15 ± 0.48	40.37 ± 0.57
*C. ovate*	30.65 ± 0.45	27.24 ± 1.05	28.25 ± 0.58
*C. portulacaria*	44.58 ± 1.59	40.14 ± 1.48	41.55 ± 2.57
*S. anacampseros*	13.96 ± 1.57	11.25 ± 1.9	10.17 ± 1.78
*S. nussbaumerianum*	28.88 ± 0.54	33.18 ± 1.24	34.25 ± 0.74
*S. sieboldii*	28.18 ± 0.24	22.05 ± 0.66	26.47 ± 0.85

**Table 3:** Compounds tentatively identified by UHPLC/QTOF–MS of *S. sieboldii* extract.

Basic Structure		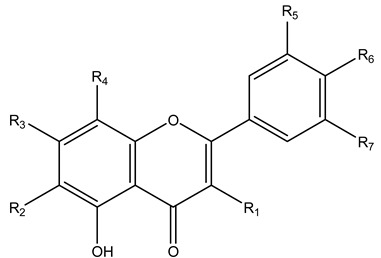					
Comp. No.	R_1_	R_2_	R_3_	R_4_	R_5_	R_6_	R_7_
9	OH	H	-O-neohesperidoside		H	OH	H
10	H	-C-β-D-Glucopyranoside	OH	H	OH	OH	H
12	H	-C-β-D-Glucopyranoside	-O-β-D-Glucopyranoside	H	H	OH	H
13	-O-α-L-rhamnopyranoside	H	OH	H	OH	OH	OH
14	H	H	-O-β-D-Glucopyranoside	-C-β-D Glucopyranoside	H	OH	H
16	H	H	OH	-C-β-D-Glucopyranoside	H	OH	H
17	-O-β-D-Glucopyranoside	H	OH	H	OCH_3_	OH	OCH_3_
18	-O- glucuronide	H	OH	H	H	OH	H
19	-O-D-xyloside	H	OH	H	OH	OH	H
20	H	OH	-O-β-D-Glucopyranoside	H	H	H	H
21	-O-α-L-rhamnopyranoside	H	-O-α-L-rhamnopyranoside	H	H	OH	H
22	OH	H	-O-β-D-rhamnopyranoside	H	OH	OH	H
23	H	H	-O-neohesperidoside	H	H	OH	H
24	OH	H	OH	H	OH	OH	OH
26	H	H	OH	H	OH	OH	H
28	OH	H	OH	H	OH	OH	H
30	H	H	OH	H	H	OH	H
31	OH	H	OH	H	H	OCH_3_	H
32	o-α-L-arabinoside	H	OH	H	H	OH	H
33	-O-α-L-rhamnopyranoside	H	OH	H	H	OH	H
35	OH	H	OH	-O-α-D-Glucopyranoside	OH	OH	H
36	OH	H	OH	H	-O-β-D-Glucopyranoside	OH	H
38	H	H	OH	-C-β-D-Glucopyranoside	OH	OH	H
39	H	H	-O-neohesperidoside	H	H	OCH_3_	H

## Data Availability

All data are available from the corresponding author upon a reasonable request.
